# Inherited Immunodeficiencies With High Predisposition to Epstein–Barr Virus-Driven Lymphoproliferative Diseases

**DOI:** 10.3389/fimmu.2018.01103

**Published:** 2018-06-04

**Authors:** Sylvain Latour, Sarah Winter

**Affiliations:** ^1^Laboratory of Lymphocyte Activation and Susceptibility to EBV infection, INSERM UMR 1163, Paris, France; ^2^Imagine Institute, Paris Descartes University, Sorbonne Paris Cité, Paris, France; ^3^Equipe de Recherche Labéllisée, Ligue National contre le Cancer, Paris, France

**Keywords:** immunodeficiencies, Epstein–Barr virus, lymphoproliferative disorders, genetic predisposition to disease, T lymphocytes, T lymphocyte activation

## Abstract

Epstein–Barr Virus (EBV) is a gamma-herpes virus that infects 90% of humans without any symptoms in most cases, but has an oncogenic potential, especially in immunocompromised individuals. In the past 30 years, several primary immunodeficiencies (PIDs) associated with a high risk to develop EBV-associated lymphoproliferative disorders (LPDs), essentially consisting of virus-associated hemophagocytic syndrome, non-malignant and malignant B-cell LPDs including non-Hodgkin and Hodgkin’s types of B lymphomas have been characterized. Among them are *SH2D1A* (*SAP*), *XIAP, ITK, MAGT1, CD27, CD70, CTPS1, RASGRP1*, and *CORO1A* deficiencies. Penetrance of EBV infection ranges from 50 to 100% in those PIDs. Description of large cohorts and case reports has refined the specific phenotypes associated with these PIDs helping to the diagnosis. Specific pathways required for protective immunity to EBV have emerged from studies of these PIDs. SLAM-associated protein-dependent SLAM receptors and MAGT1-dependent NKG2D pathways are important for T and NK-cell cytotoxicity toward EBV-infected B-cells, while CD27–CD70 interactions are critical to drive the expansion of EBV-specific T-cells. CTPS1 and RASGRP1 deficiencies further strengthen that T-lymphocyte expansion is a key step in the immune response to EBV. These pathways appear to be also important for the anti-tumoral immune surveillance of abnormal B cells. Monogenic PIDs should be thus considered in case of any EBV-associated LPDs.

The Epstein–Barr virus (EBV) is a gamma-herpes family virus that infects most of humans with a marked tropism for B lymphocytes. EBV is an oncogenic virus known to be the causative agent of several types of neoplasms, including B, T, and NK cell lymphomas, nasopharyngeal and gastric carcinomas, and Epstein–Barr virus-associated smooth muscle tumors (EBV-SMT). 110,000–200,000 cancer cases per year are attributable to EBV worldwide (1).

The reservoir of EBV is strictly human. After the age of 35 years, the incidence of the infection in the general population is more than 90%. The primary infection occurs in the oropharynx where EBV virions infect epithelial cells and B lymphocytes *via* the CD21 molecule. During the primary infection, EBV drives the activation and the expansion of latently infected B lymphoblasts (2, 3). These proliferating B cells express EBV latent growth-transforming genes that establish EBV persistence (latency III program) and are mainly eliminated by specific CD8^+^ T cells that strongly expand during the immune response. Innate cytotoxic lymphocytes like NK cells, γδ T cells, and iNKT cells, specifically early differentiated KIR-negative NK cells and Vγ9Vδ2 T cells, are also thought to play an important role in the early phase of the primary infection by recognition of lytically and latently EBV-replicating cells, respectively (2, 4, 5). Some EBV-infected B cells escape to the immune response by downregulating latent genes expression (latency 0 program) and acquire a memory phenotype, becoming invisible to the immune system and establishing a reservoir for EBV. Subsequent stimulations of these EBV-containing “reservoir” memory B cells will lead to reactivation of EBV from latency into the lytic cycle, thus promoting infections of new B cells and their expansion. Ultimately, EBV-transformed lymphoblasts can lead to lymphoma. In some very rare cases, EBV can also infect T cells and NK cells. This peculiar profile of infection is rather observed in Asian and South American populations and is associated with a chronic viremia, infiltration of organs with by EBV-positive lymphocytes, and life-threatening lymphoproliferative disorders (LPDs) including hemophagocytic syndrome or/and EBV-positive T/NK cell lymphoma. The mechanisms underlying the pathogenesis of this infection are not clearly known, as well as its genetic determinants that are thought to be oligogenic or polygenic (6, 7). This unusual EBV infection will not be covered in this review.

The first encounter with EBV usually happens during infancy and adolescence by oral transmission and is largely asymptomatic. However, in some immunocompetent individuals particularly during adolescence, primary infection causes infectious mononucleosis (IM), a self-limiting lymphoproliferative disease clinically characterized by fever, sore throat, body aches, swollen lymph nodes, and general fatigue (3). The lymphoproliferation consists of a robust and sustained expansion of CD8^+^ T cells and infected B cells reflecting a strong immune response to the virus. Notably, CD8^+^ EBV-specific T cells can represent more than 40% of circulating T cells in some subjects (8).

In immunocompromised individuals, reactivations of EBV and persistence of proliferating latent growth-transforming EBV-infected B cells are associated with severe pathologies that can have fatal outcome. Those include hemophagocytic lymphohistiocytosis (HLH), also termed virus-associated hemophagocytic syndrome, non-malignant B-cell LPDs, and B-cell lymphomas including Hodgkin’s lymphomas and non-Hodgkin’s lymphomas such as Burkitt’s lymphoma and diffuse large B-cell lymphoma (DLBCL) (1). Such disorders defined as posttransplant lymproliferative disorders are often observed in patients with organ transplantation under immunosuppressive treatment. Similarly, HIV-infected patients with acquired immunodeficiency syndrome (AIDS) often experience lymphoproliferation disorders driven by EBV, that represent one of the most frequent cause of death in patients with AIDS (9). Those observations highlight that reactivations of EBV from latently EBV-infected B cells occur frequently in normal individuals throughout life and need to be tightly controlled by the adaptative immune response.

Beside acquired forms, several inherited combined immunodeficiencies (CIDs) leading to a particular susceptibility to EBV infection and to develop EBV-driven diseases have been identified over the last 20 years (10–12). Those genetic defects include mutations in *SH2D1A, ITK, MAGT1, CTPS1, CD27, CD70, CORO1A*, and *RASGRP1* (Table 1). In these genetically determined forms, the penetrance of the EBV susceptibility is high with more than 50% patients having presented at least one severe episode of EBV-driven LPD including Hodgkin and non-Hodgkin lymphomas (Table 2). However, a number of carriers of these gene defects can also experience other severe viral infections caused by CMV, VZV, HSV, HHV-6, or HPV. This is particularly true for CTPS1 and CORO1A deficiencies since patients often presented VZV and HPV infections, respectively. Bacterial infections, in particular recurrent lung infections are noticed in a number of patients and can be the initial clinical presentation. This may be related to the hypogammaglobulinemia and/or dysgammaglobulinemia associated with low number of CD27^+^ memory B cells that are frequently observed in these defects. These phenotypes likely result or have been proven to result from abnormalities in T-cell help to B cells due to defects in T-helper cell maturation and/or activation. Intrinsic defects in B cell development and function may also contribute directly to the hypo/dysgammaglobulinemia, like in RASGRP1, CTPS1, CD27 deficiencies, for which there is clear evidence that these genes are directly involved in B-cell development and/or function. However, these B-cell defects do not to interfere with the ability to EBV to infect B cells and its oncogenic activity. CD4^+^ T cell lymphopenia and/or decrease naïve T cells is also a frequent phenotype, which can be considered as an immunological hallmark of a T-cell defect (13, 14). NK cells and iNKT cells are also often found to be diminished or absent. Finally, several of these deficiencies are associated with very unique features, independently of the high risk to develop EBV-driven lymphoproliferations, helping to the diagnosis, such as autoimmunity, inflammatory bowel disease (IBD), lung involvement, and neurological disorders that develop in RASGRP1, XIAP-, ITK-, and CORO1A-deficiency, respectively (Table 1).

**Table 1:** Lymphoma subtypes in primary immunodeficiencies associated with high susceptibility to develop EBV-driven lymphoproliferative diseases.

Mutated gene number of patients (*n*)	Age of onset (years)	EBV-associated diseases	Infections		Other clinical features	Immunological features			Outcome	Defective pathways/functions
						T-cells	B-cells	NK-cells		
**SH2D1A (XLP-1)** *n* > 100	0.5–40	SIM/HLH 50–60% (neurological inv. 25%) B lymphoma 25–30% Vasculitis 3–4%	**EBV 60–70%** HHV-6 (1) HHV-7 (1) LCMV (1)	Lung infections 15% Diss. aspergillosis (1) *E. coli* sepsis (1)	Aplastic anemia 3–4% EBV-negative lymphoma EBV-negative vasculitis	Absence iNKT	Dys-γ, hypo-γ 50%	↓ NK	HSCT 10–40% Mortality 20–50%	SLAMR/SAP pathway (T and NK cytotoxicity and AICD)
**XIAP (XLP-2)** *n* > 100	0.5–40	SIM/HLH 50%	**EBV 35–40%** CMV 10–20% HHV-6 (1)		IBD 25–40% Inflammatory disorders 5% (uveitis, skin abcesses, etc.) Cholangitis 1–2%	↓ MAIT ↓ iNKT	Hypo-γ 15–20%		HSCT 10–30% Mortality 5–30%	Excess of apoptosis (AICD, TRAIL-R, Fas) NOD1/2 signaling/function
**ITK** *n* = 13	2.5–18	LPD/B lymphoma 13/13 HLH 1/13 SMT 1/13	**EBV 13/13** CMV 1/13 VZV 1/13 BK-virus 2/13	Lung infections 9/13 PCP 2/13	Lung involvement 11/13 Kidney involvement 3/13 Al cytopenias 3/13	↓ CD4^+^ 8/13 ↓ iNKT 5/5	Hypo-γ 8/13		HSCT 4/13 Death 7/13	TCR induced calcium flux T-cell proliferation
**MAGT1** *n* = 11	3–45	LPD/B lymphoma 7/11	**EBV 11/11** HSV 2/11, VZV 1/11 JC virus (PML) 1/11 HHV-8 (KS) 1/11	Lung infections 9/11	Al cytopenias 3/11	↓ CD4^+^ ↓ NKG2D	Dys-γ, hypo-γ 8/11 Lymphocytosis		HSCT 2/11 Death 3/11	NKG2D-dependent cytotoxicity
**CORO1A** *n* = 9	0.5–7	LPD/B lymphoma 5/9	**EBV 5/9** HPV 4/9, VZV 5/9 HSV 2/9 Parvovirus B19 1/9	Lung infections 7/9 Cutaneous leprosy 1/9 Visceral leishmaniasis 1/9	Neurological involvement (cognitive impairment) 3/9	↓ CD4^+^ 8/9 ↓ MAIT 1/1 ↓ iNKT 1/1	High IgE level 4/5		HSCT 2/9 Death 4/9	Actin regulation T-cell survival NK cytotoxicity
**CD27** *n* = 18	1–22	LPD/B lymphoma 12/18 T-cell lymphoma 1/18 SIM/HLH 5/18 Meningitis 1/18	**EBV 18/18** VZV 2/18 CMV 1/18	Lung infections 4/18 Gram-positive sepsis 1/18 Giardiasis 1/18	Aplastic anemia 1/18 Uveitis 5/18 Oral anal ulcers 5/18	↓ iNKT 3/10	Hypo-γ 13/18	↓ NK	HSCT 4/18 Death 3/18	CD27–CD70 pathway (T-cell proliferation) NK cytotoxicity
**CTPS1** *n* = 12	0–5	LPD/B lymphoma 5/12 SIM 5/12	**EBV 11/12** VZV 6/12 Norovirus 3/12 CMV 2/12, HHV-6 2/12	Lung infections 7/12 Meningitis 3/12	Eczema 2/12	↓ MAIT ↓ iNKT	↓ lgG2 5/5 ↓ CD27^+^ B-cells	↓ NK	HSCT 9/12 Death 3/12	*De novo* pyrimidine synthesis T- and B-cell proliferation
**RASGRP1** *n* = 6	5–12	LPD/B lymphoma 4/6 SMT 2/6	**EBV 5/6** HSV 1/6 HPV (EV) 1/6 CMV 1/6	Lung infections 5/6 Diss. tuberculosis 1/6 LN tuberculosis 1/6 PCP 1/6	Al cytopenias 3/6 EBV-negative LPD 2/6	↓ CD4^+^ 4/6 ↓ MAIT 2/2 ↓ iNKT 2/2	Hypo-γ 1/6 Hyper-γ 2/6 ↓ CD27^+^ B-cells 2/4	↓ NK 3/6	HSCT 2/6 Death 2/6	MAPK pathway (ERK1/2, T-, B-cell proliferation) Actin/cytoskeleton dynamics NK cytotoxicity
CD70 *n* = 6	1–5	LPD/B-cell lymphoma 5/6	EBV 6/6 VZV 1/6	Lung infections 3/6	PFAPA 1/6 Hypersensitivity to mosquito bites 1/6	↓ MAIT 1/1 ↓ iNKT 1/1	Hypo-γ 5/6 ↓ CD27^+^ B-cells 3/6	HSCT 1/6 All alive	CD27–CD70 pathway (T-cell proliferation)	Decreased 2B4 and NKG2D on memory T CD8^+^

*SIM, severe infectious mononucleosis; HLH, hemophagocytic lymphohistiocytosis; LPD, lymphoproliferative disorder; inv., involvement; Diss., disseminated; PCP, pneumocytosis pneumoniae; IBD, inflammatory bowel disease; Al, autoimmunity; Dys-γ, dysgammaglobulinemia; Hypo-γ, hypogammaglobulinemia; KS, Kaposi sarcoma; PFAPA, perdiodic fever, aphtous stomatitis, pharyngitis and cervical adenitis; EV, epidermodysplasia verruciformis; SMT, smooth muscle tumor; PML, progressive multifocal leukoencephalopathy; HSCT, hematopoietic stem cell transplantation; LN, lymph node; EBV, Epstein–Barr virus; LCMV, lymphocytic choriomeningitis virus; CMV, cytomegalovirus; HHV, human herpes virus; VZV, varicella zoster virus; MAIT, mucosal-associated invariant T; AICD, activation-induced cell death; TCR, T-cell receptor; SAP, SLAM-associated protein; SLAMR, SLAM receptor*.

**Table 2:** Lymphoma subtypes in primary immunodeficiencies associated with high susceptibility to develop EBV-driven lymphoproliferative diseases.

Mutated gene number of patients/total	Hodgkin lymphoma (subtype)	B-cell NHL			T-cell lymphoma
		DLBCL	Burkitt lymphoma	Not specified	
**SH2D1A** 25–30%		30–40%: abdominal (40–50%), cervical (30–40%), spinal (10–20%)	40–60%: abominal (50–60%), cervical (20%)	20–30%	1 patient (CNS)
**ITK** 9/13	6 patients (1 mixed cellularity)	1 patient	1 patient	1 patient	
**MAGT1** 5/11	2 patients	1 patient	1 patient	1 patient	
**COR01A** 4/9		4 patients			
**CD27** 6/18	3 patients (2 scleronodular and 1 mixed cellularity)			2 patients	1 patient
**CTPS1** 2/12				2 patients (CNS 2/2)	
**RASGRP1** 4/6	2 patients (1 scleronodular and 1 mixed cellularity)			2 patients	
**CD70** 4/6	4 patients (1 scleronodular and 1 mixed cellularity)				

*CNS, central nervous system; NHL, non-Hodgkin lymphoma; DLBCL, diffuse large B-cell lymphoma; EBV, Epstein–Barr virus*.

Over the last two decades, these inherited defects have represented very unique *in natura* models to decipher the immune response to EBV. Molecular and genetic characterization of these disorders and their study have revealed several unexpected and novel pathways required for an efficient immunity to EBV, but also more generally involved in cancer immune surveillance of B lymphocytes, and adaptative immune responses.

## SH2D1A/SLAM-Associated Protein (SAP) Deficiency

SH2D1A/SAP deficiency (also known as the X-linked lymphoproliferative syndrome type 1-XLP-1 or Purtilo syndrome) is caused by hemizygous mutations in the X-linked gene *SH2D1A* (15–19). More than 100 patients with hemizygous deleterious mutations in *SH2D1A* have been reported, but if considering the literature before the discovery of the gene, this is probably more than 200 cases (20–22). Clinical features of XLP-1 are EBV-driven fulminant or severe mononucleosis with all the clinical features of HLH including fulminant hepatitis, hepatic necrosis, bone marrow hypoplasia, neurological involvement in 20–30% of cases, hypogammaglobulinemia (50%), and B-cell lymphoma (25–30%) which often have abdominal localization. Few other rare phenotypes can be also observed such as vasculitis (2–5%), aplastic anemia (2–5%), and chronic gastritis (18, 23–26). All phenotypes can occur independently and some develop without any evidence of EBV infection (10–20%) or prior to EBV infection like HLH, hypogammaglobulinemia, lymphoma, aplastic anemia, and vasculitis (24–26). Some rare cases of T-cell lymphoma have also been reported (23, 27). However, a significant proportion of patients initially presented with combined variable immunodeficiency (CVID) associated with severe recurrent bacterial infections including disseminated aspergillosis and *E. coli* sepsis (28–31). Only five cases of other severe viral infections by HHV-6, HHV-7, and LCMV or HLH without EBV trigger have been reported in the recent literature (22, 26, 32, 33). This highlights that SAP has a very unique role in immunity to EBV, when it is not required for other viral infections except in very rare cases.

*SH2D1A* encodes a small adaptor protein of 128 amino acids named SAP, uniquely expressed in T and NK cells. SAP is only made of a unique SH2 domain that specifically binds to the intracytoplasmic domain of receptors of the SLAM family (SLAMF), which are self-ligands and are involved in homotypic cell–cell interactions with the exception of 2B4 (also known as CD244 or SLAMF4) that binds CD48 (34–36). On the one hand, SAP functions as a real adapter protein by its ability to recruit the tyrosine kinase FynT to SLAMF receptors, thus coupling those to downstream signaling pathways. On the other hand, SAP acts also as a blocker protein by inhibiting the recruitment of SH2-containing phosphatases to SLAMF (15, 35, 37–39). Early studies have documented NK-cell cytotoxicity defects toward EBV-infected B cells in XLP-1 patients (40, 41). Further investigations revealed that these defects involved two SLAM receptors, 2B4 and SLAMF6 (also known as NTB-A or Ly108) which have the capacity to trigger and activate NK- and CD8^+^ T-cell cytotoxicity toward EBV-infected B cells or transformed B cells when SAP is normally expressed (42–46). B-cell lymphomas and EBV-infected B cells are known to express high levels of ligands for SLAMF-R, including CD48 the ligand of 2B4 (47). This expression might signal abnormal “dangerous” proliferating B cells to NK cells and T lymphocytes. Following studies demonstrated that the stimulatory function of 2B4 and SLAMF6, which is dependent of FynT was not only lost in the absence of SAP but also shifted toward an inhibition of other stimulatory pathways of cell cytotoxicity in NK- and CD8^+^ T-cells (43, 48, 49). Additional findings in mice indicated that this inhibitory effect was independent of FynT, but depends of the blocking activity of SAP, and leads to decreased conjugate formation between CD8^+^ T or NK cells and SLAMF-expressing target cells like B cells (50, 51). In this context, other activation pathways of cytotoxicity such as NKG2D may not compensate for the defective function of 2B4 and SLAMF6. The inhibitory activity of 2B4 and SLAMF6 in the absence of SAP may have a trans-inhibitory effect on activating receptors (such as NKG2D and/or killer cell activating receptors) when co-engaged at the cytotoxic synapse.

Importantly, NK-cell and CD8^+^ T-cell cytotoxicity toward other APCs or target cells than B cells including non-hematopoietic cells or/and SLAMF-negative target cells was preserved, and even augmented for NK-cell cytotoxicity (47, 52, 53). Recent studies in mice indicate that this excessive NK-cell cytotoxicity is dependent of a role of SAP and SLAMF6 in NK cell licensing/education (52). In the absence of SAP, NK-cell responsiveness toward SLAM-negative cells is increased. In XLP-1 patients, increased NK cytotoxicity toward the SLAMF-negative target cells K562 was documented (54), associated with increased CD16- and NKp46-dependent cytotoxicity (52). Thus, extra-hematopoietic manifestations associated with HLH seen in XLP-1 patients, like extensive liver damage may be caused by this increased NK-cell cytotoxicity toward non-hematopoietic cells.

Beside its function in NK- and CD8^+^ T cell-cytotoxicity, SAP is also important to limit CD8^+^ T-cell expansion. SAP-deficient T cells both in mice and human exhibited increased survival due to impaired activation-induced cell death (AICD), an important pathway involves in the contraction of the pool of Ag-specific T-cells during immune responses (55, 56). The positive role of SAP on AICD is dependent of SLAMF6 that delivers signals for the expression of the pro-apoptotic molecules Bim and FasL (55). In concert, these distinct defects certainly contribute to the inability of EBV-specific T cells to eliminate EBV-infected B cells and to their uncontrolled expansion and activity during primary infection leading to fulminant mononucleosis or HLH. The fact that patients also frequently develop non-EBV B cell lymphoma argues for an important role of the SLAM–SAP pathway in the immune surveillance of B cells.

## XIAP Deficiency

XIAP deficiency (also known as the X-linked lymphoproliferative syndrome type 2/XLP-2) is caused by hemizygous mutations in the *XIAP* gene coding the X-linked Inhibitor of Apoptosis Protein XIAP (54, 57). XIAP is an anti-apoptotic molecule member of the inhibitor of apoptosis protein family (IAP). XIAP is a potent inhibitor of program cell death, but it is also required for the function of (NOD)-like pattern recognition receptors (NLRs) NOD 1 and 2 and the regulation of the inflammasome activity of NLRP3 (58–61). More than 100 patients have been now reported with a XIAP deficiency. Initially reported in 2006 in a cohort of patients with a clinical phenotype close to that of SH2D1A-deficient patients (XLP-1), XIAP deficiency was further denominated XLP-2, since *XIAP* is located in the immediate vicinity of *SH2D1A* on the X chromosome (57). However, during the last 10 years, there were cumulative observations that the two diseases differ by many aspects, in particular, there is no evidence that they are functionally related. The main clinical phenotypes of the XIAP deficiency are the susceptibility to develop HLH in the context of EBV infection (36%), the recurrent splenomegaly corresponding to a minimal form of HLH (57%), and the IBD (26%) with features of Crohn’s disease (57, 62, 63). The HLH is often less severe than in the XLP-1 deficiency with very rare neurological involvement (24). Some patients also developed variable auto-inflammatory symptoms like uveitis, arthritis, skin abscesses, erythema nodosum, and nephritis (62, 63). Thus, today although the susceptibility to EBV remains one important and severe clinical manifestation of the XIAP deficiency, more than half of the patients never experienced a peculiar EBV susceptibility (57, 62). XIAP-deficient patients also never experience lymphoma in contrast to patients with SAP deficiency, likely related to the anti-apoptotic function of XIAP, which may protect patients from cancer, and XIAP is now considered as a promising therapeutic target for cancer treatment (64).

T lymphocytes from XLP-2 patients have been shown to exhibit increased AICD in response to T-cell receptor (TCR) activation and increased apoptosis to FAS/CD95 and TRAIL-R stimulations (54). On the other hand, monocytes from patients displayed impaired production of cytokines and chemokines (TNF-α, IL-10, IL-8, and MCP-1) to stimulation with NOD2 ligands (63, 65). Although the mechanisms of EBV-driven HLH in XIAP deficiency remains unclear, it is proposed that excessive AICD might compromise the expansion and proliferation of activated EBV-specific T cells like in CTPS1, CD27, or CD70 deficiencies (see below)(58). In this setting, accumulation of apoptotic cells and persistence of EBV-infected B cells could result in abnormal inflammation amplified by impaired inflammasome regulation and/or defective NOD1/2 activation of myeloid cell populations. In concert, T lymphocytes and myeloid defects may contribute to EBV-driven HLH in XIAP-deficient patients.

## ITK Deficiency

ITK deficiency is an autosomal recessive disorder caused by bi-allelic mutations in *ITK*. Until now, 13 patients from 8 families have been reported in the literature (66–72). All experienced EBV-associated recurrent non-malignant LPD or malignant B-cell lymphoproliferations including Hodgkin lymphoma in five, and one develop an EBV-SMT (69). B-cell lymphoproliferations have often a pulmonary localization and more than 50% also develop pulmonary infections. ITK (IL-2-inducible tyrosine kinase) is a well-characterized protein tyrosine kinase of the TEC/BTK family specifically expressed in T lymphocytes and NK cells. ITK is involved in TCR signaling by its capacity to phosphorylate and activate the PLC-γ1, a key enzyme that stimulates Ca^++^ fluxes through the production of IP3, both being critical second messengers of T-cell activation (73, 74). ITK has been also involved in CXCR4 signaling (75). Mice studies demonstrated the importance of ITK in immunity particularly in CD4^+^ T-cell responses (73, 76). A recent report showed that ITK is also required for efficient CD8^+^ T-cell responses in mouse (77). In the absence of ITK, CD8^+^ T-cell expansion and maturation into cytolytic effector T cells is impaired leading to decreased CD8^+^ T-cell cytotoxic responses. Very few studies have been made to characterize ITK-deficient T cells from patients, albeit-derived T-cell lines from ITK-deficient patients have been obtained and studied showing decreased Ca^++^ mobilization (67). Defective T-cell proliferation in response to TCR engagement was documented in one patient (69). Although the exact mechanism(s) underlying EBV susceptibility in ITK deficiency need to be established, the recent study of CD8^+^ T cells from ITK-deficient mice (77), strongly supports that in T cells from ITK-deficient patients, TCR activation signals are impaired resulting in defective expansion and maturation of EBV-specific CD8^+^ T cells like in CD70 and CD27 deficiencies (see below).

## MAGT1 Deficiency

MAGT1 deficiency also termed XMEN disease (for X-linked immunodeficiency, magnesium defect, EBV infection, and neoplasia syndrome) is caused by hemizygous mutations in *MAGT1*. To date, 11 male patients with MAGT1 deficiency have been identified and all developed susceptibility to EBV infection with chronic viremia and B-cell lymphomas including Hodgkin and DLBCL (78–82). In one patient, the initial clinical presentation was a HHV-8-associated Kaposi sarcoma (80). One particular trait of this disease is the B-cell lymphocytosis associated with a CD4 lymphopenia (83). *MAGT1* encodes a ubiquitously expressed transmembrane Mg^++^ transporter involved in the maintenance of free basal intracellular Mg^++^ pools. However, MAGT1 also associates with the *N*-oligosaccharyl transferase complex, and therefore may have a role in protein N-glycosylation (84). Following the discovery of the MAGT1 immunodeficiency, MAGT1-dependent Mg^++^ influx was documented in T cells upon TCR engagement. Importantly, this Mg^++^ mobilization was shown to be involved in PLC-γ1 activation and subsequent dependent Ca^++^ influx (78). Notably, all these events were markedly impaired in activated MAGT1-deficient T cells in response to TCR, but T-cell proliferation has been considered to be normal or diminished (85). Based on these findings, it was proposed that MAGT1 might be necessary to activate PLC-γ1 possibly by acting on ITK. In a recent study, intriguingly, Ca^++^ mobilization was found to be only moderately decreased and delayed in T cells from a patient carrier of a deletion encompassing *MAGT1* (80). Along these lines, an other recent report showed that MAGT1-deficient T cells from mice exhibited normal calcium flux upon TCR activation, while calcium flux was impaired in B cells in response to BCR stimulation (86). The discrepancy between these recent observations and earlier studies is not known. In any case, MAGT1 was further shown to be required for expression and function of NKG2D and its signaling adapter DAP10 (79). NKG2D is an activating NK-cell receptor expressed on NK cells, γδ T cells, and CD8^+^ T cells, that recognizes MHC class I-homologous proteins induced by cellular stress in response to infection or neoplasia. Importantly, ligands of NKG2D are upregulated on EBV-infected B cells and EBV-associated lymphoproliferations (87, 88). Consistent with defective NKG2D expression, MAGT1-deficient CD8^+^ T cells displayed impaired cytotoxic activity against autologous EBV-transformed B cells. Importantly, magnesium supplementation treatment *in vivo* and *in vitro* restored basal intracellular Mg^++^ concentration, NKG2D expression, cell cytotoxicity, and immunity to EBV in MAGT1-deficient patients (79). Defective N-glycosylation of NKG2D associated with increased ubiquitinylation leading to accelerated protein turnover might be considered as the main mechanism explaining the impaired NKG2D function. Thus, these observations highlight the essential role of the NKG2D pathway in immunity to EBV, albeit the exact role of MAGT1 in TCR signaling remains to be clarified.

## CD27 Deficiency

This deficiency is caused by bi-allelic mutations in *CD27* that encodes a protein belonging to the super family of TNF receptors (TNFSFR), also known as TNFSFR7. Until today, 18 patients have been reported and all developed EBV-associated LPDs including malignant B-cell proliferations, Hodgkin’s lymphoma, B lymphoma, and few patients developed HLH triggered by EBV (89–92). Some patients also experienced inflammatory symptoms such as uveitis and oral ulcers. CD27 binds to CD70 (also named TNFSF7), a member of the TNF superfamily ligands. CD27 is highly expressed by T cells including resting T cells and a small fraction of B cells corresponding to memory B cells (93–95). CD27 is a co-stimulatory molecule of T-cell activation and CD27–CD70 interactions in mice have been shown to enhance T-cell survival, effector functions, and memory T-cell expansion, in particular CD8^+^ T cells during anti-viral immune responses (96, 97). Insights to the mechanism underlying the high susceptibility to EBV in CD27 deficiency was recently given by the identification of CD70-deficient patients (see below).

## CD70 Deficiency

Five patients with homozygous deleterious mutations in *CD70* have been reported in 2017 and one in 2018 (91, 98, 99). Five out of six patients developed EBV-Hodgkin’s lymphoma and recurrent B-cell lymphoproliferations, and one initially presented clinical signs evoking a Behçet-like syndrome and had uncharacterized viral encephalitis. All patients had dysgammaglobulinemia and two patients developed other infections. By many aspects CD70-deficiency appears to be a phenocopy of the CD27 deficiency. In peripheral blood mononuclear cells, CD70 is only expressed by a very small fraction of B cells (91), but its expression is strongly upregulated on activated B cells and EBV-infected B cells during primary infection in tonsils of patients with IM (91). B-cell lymphomas and several other cancers such as solid carcinomas are also known to express CD70 (94). Izawa et al. demonstrated that CD70 expression on EBV-infected B cells drives the expansion of EBV-specific cytolytic T cells *via* a TCR-CD27-dependent co-stimulation. When CD70 is absent on EBV-infected B cells, or CD27 on T cells, EBV-specific T cells failed to expand leading to reduced cytotoxicity responses toward EBV-infected B cells (91). Decreased expression of 2B4 and NKG2D on memory CD8^+^ T cells of CD70-deficient patients was also noticed and may also contribute to the inability of T cells to eliminate EBV-infected B cells (98). These findings demonstrate that the CD70–CD27 axis represents a key component of the protective immunity to EBV. Furthermore, the implication of the CD27–CD70 axis in anti-tumoral immune surveillance of abnormal B cells is supported by the observations that *CD70* is often found somatically mutated in B lymphomas, perhaps to escape to the immune surveillance (91).

## CTPS1 Deficiency

CTPS1 deficiency is an autosomal recessive immunodeficiency caused by a unique homozygous deleterious mutation in *CTPS1* with a founder effect in the population of the North West of England. Until now, 12 patients have been reported and all but 1 presented EBV susceptibility including severe infectious mononucleosis, LPD, and B-cell lymphoma, which was the initial clinical presentation in 40% of cases (100–102). Half of them also developed other viral infections including CMV, VZV, and HHV-6. Some also experienced recurrent bacterial infections with *Haemophilus influenza, Streptococcus pneumonia*, and/or *Neisseria meningitis*. *CTPS1* codes for the CTP synthetase or synthase 1, a key enzyme of the *de novo* synthesis of the CTP nucleotide, which is the limiting nucleotide in cells (103). CTP is a critical precursor in the metabolism of nucleic acids. CTP is produced by two pathways, a salvage pathway and a *de novo* synthesis pathway. The salvage pathway utilizes cytidine, a degradation product from nucleic acids. The “*de novo*” CTP synthesis is dependent of two enzymes CTPS1 and CTPS2, which catalyze ATP-dependent amination of UTP to CTP with ammonia (-NH3) transfer from hydrolyzed glutamine. In normal tissues, CTPS activity is rather low, while it is high in proliferating cells like cancer cells including lymphoma. Importantly, CTPS1 is very low in resting T cells and is rapidly and strongly upregulated in response to TCR stimulation (100). In CTPS1-deficient patients, the proliferation of T cells in response to TCR engagement is markedly impaired, while other T-cell responses including cytokines production, AICD, and cytotoxicity are not affected, and T cells normally proliferate in response to IL-2. Addition of CTP or cytidine in the culture medium restored T-cell proliferation of activated T cells. CTPS1 expression is also upregulated in activated B cells. However, the role of CTPS1 in B cells may be less important than in T cells as patients developed rather infections associated with a T-cell defect and the absence of CTPS1 has no effect on proliferation of EBV-infected B cells and their transformation by EBV. The discovery of the CTPS1 deficiency emphasizes the importance of T-cell expansion during anti-viral responses, specifically in primary infection to EBV, since 40% T cells of circulating can be specific to EBV (8).

## CORO1A Deficiency

Deficiency in the actin regulator CORO1A (Coronin-1A) has been identified in nine patients (104–108). Patients presented with severe infections, and five developed EBV-driven B cell lymphoma. Four patients had severe mucocutaneous-immunodeficiency manifestations including epidermodysplasia verruciformis-HPV (EV-HPV) (104, 108). Three patients also exhibited neurological abnormalities including autism-like symptoms. Patients with CORO1A deficiency are characterized by a profound T cell lymphopenia with strongly decreased or nearly absent naïve cells associated with defective thymic output. CORO1A-deficient T cells from patients showed increased spontaneous *in vitro* apoptosis, delayed ERK1/2 activation, increased filamentous actin, but normal or reduced T cell proliferation to mitogens and antigens and normal calcium flux and cytotoxicity. It is proposed that CORO1A deficiency is primarily a T-cell immunodeficiency caused by impaired thymic egress, migration, and survival of mature T cells, thereby affecting lymphocyte homeostasis, repertoire selection, and lineage commitment (109). CORO1A belongs to the family of Coronins that are evolutionarily conserved intracellular actin-binding proteins expressed at high level in most of leukocyte populations (110). In T cells, CORO1A has been shown to be a negative regulator of branched F-actin formation and be required for chemokine-mediated migration and lymphocyte survival given the fact that accumulation of F-actin is known to be toxic for cells (108, 111). However, CORO1A in T cells has been also involved in a variety of pathways including TCR and TGF-β signaling and immunologic synapse (IS) formation (112–115). Along these lines, defective NK cell degranulation was reported in one patient in association with increased density of F-actin at the cytotoxic synapse (113). Neurological abnormalities found in patients are likely explained by the role of coronin-1A in neurodevelopment that has been reported in mice (116). Finally, Coronin 1A was recently shown to be required for neutrophil trafficking. These observations suggest that the immune defects in patients may not only be restricted to T cells (117). While it is not clearly established why patients with CORO1A deficiency are susceptible to EBV, it is very likely that the poor T-cell survival may result in defective expansion of EBV-specific CD8^+^ T-cells leading to impaired control of EBV-infected B cells. Interestingly, four patients developed EV-HPV, which could bring closer CORO1A deficiency to primary immunodeficiencies (PIDs) associated with frequent and extensive HPV infections, especially those such as MST1/STK4 and DOCK8 deficiencies, that are characterized by CD4 lymphopenia, defective T cell migration, and/or abnormal F-actin polymerization and IS formation (118).

## RASGRP1 Deficiency

RASGRP1 deficiency is caused by bi-allelic mutations in *RASGRP1*. So far, three different null homozygous mutations have been identified in four patients having developed severe EBV-driven LPDs including two Hodgkin lymphoma. One had also an EBV-SMT (119–121). *RASGRP1* codes for a diacylglycerol-regulated guanidine exchange factor preferentially expressed in T and NK cells (122, 123), which acts as an activator of the small G protein RAS and the downstream RAF-MEK-ERK kinases cascade (also known as the MAP kinases pathway). In T lymphocytes, RASGRP1 is the main activator of the MAP kinases pathway (124, 125). RASGRP1-deficient T cells showed impaired ERK/MAPK activation and decreased T-cell proliferation in response to mitogens and antigens (119, 120). In the first report, RASGRP1-deficient T cells were also shown to have diminished cell cytotoxicity and migration capacity (120). NK cells also exhibited decreased cell cytotoxicity. A direct role of RASGRP1 in cytoskeletal dynamics during exocytosis of lytic granules in NK and T cells and in T-cell migration is suggested by its ability to interact with the dynein light chain DYNLL1 and to activate RhoA, respectively (120). This role may explain the impaired cytotoxic responses seen in RASGRP1-deficient T and NK cells. In a recent report by Winter et al., RASGRP1-deficient NK cells and CD8^+^ T cells were nonetheless found to have normal degranulation when stimulated (119). The discrepancy between these studies is not known. However, in the different reports, low numbers of NK cells were consistently noticed in RASGRP1-deficient patients that might contribute to the decreased NK cell cytotoxicity. Winter et al. further analyzed the possible mechanisms underlying the EBV susceptibility in RASGRP1 deficiency and showed that RASGRP1-deficient T cells failed to expand normally (119). In particular, RASGRP1-deficient T cells had impaired CD27-dependent proliferation toward CD70-expressing EBV-transformed B cells, a critical pathway to expand EBV-specific T cells (see above). Interestingly, the impaired proliferation of activated RASGRP1-deficient T cells correlated with their inability to upregulate CTPS1 protein expression, suggestive of a role of RASGRP1/MAPK pathway in CTPS1 expression, but also in other factors involved in T-cell proliferation, as CTP or cytidine failed to restore impaired proliferation of RASGRP1-deficient T cells (119).

Two *RASGRP1* heterozygous compound mutations with no effect on RASGRP1 protein expression have been also recently identified in two siblings with multiple fungal, bacterial, and viral infections including EBV and CMV, and both also developed autoimmunity signs evoking autoimmune lymphoproliferative syndrome (126). Along these lines, RASGRP1-deficient mice developed autoimmune LPD resembling systemic lupus erythematous when getting older (127, 128) and RASGRP1 is considered as a risk locus for autoimmunity (129–131). T cells from the two patients showed defective TCR activation associated with impaired proliferation and AICD. However, complementation experiments with wild-type RASGRP1 were unsuccessful, leaving the possibility that other or additional genetic events contribute to this particular phenotype and/or these mutations do not behave as loss-of-function mutations. These observations could suggest a genotype–phenotype correlation.

## Pathways in the Control of EBV Infection

**Figure 1:**
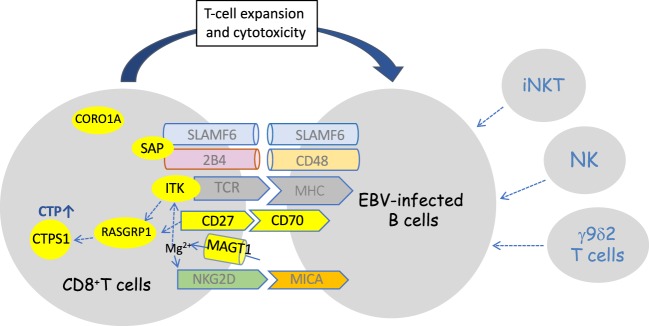
Schematic representation of defective identified pathways in immunodeficiencies predisposing to high susceptility to Epstein–Barr virus (EBV)-driven B-cell lymphoproliferative diseases. Defective components in the pathways are in yellow. Three non-redundant pathways have been shown to be impaired: the SLAMF6/2B4/SLAM-associated protein (SAP) pathway, the CD70/CD27 pathway, and the NKG2D/MICA/MAGT1 pathway. At some point, several of these pathways may converge toward distal effector molecules such as RASGRP1 and CTPS1 that are required for expansion of T cells. The connection of CORO1A to these pathways is not known. Besides the essential role of CD8^+^ T cells, NK and iNKT cells which are often decreased in these primary imunodeficiencies may play a role in the immune response against EBV, particularly during the early phase of the primary infection. Vγ9δ2 T cells are also thought to play an important role in the primary infection by recognition of latently EBV-replicating B cells.

Several key pathways required for an efficient immunity to EBV have emerged from studies of these genetic disorders (11). Interestingly, these pathways are involved in the cell–cell interaction and cross-talk between T and B cells and appear to play a critical role in the immune surveillance of B cells by T cells, which is consistent with B cells as the privileged target of EBV infection and the reservoir of EBV. These pathways implicate pairs of receptor–ligand expressed by T and B cells, respectively (Figure 1). The best known and in depth studied is the SLAMR–SAP pathway, which is defective in the SAP deficiency/XLP-1 syndrome and mainly involves two SLAM receptors, 2B4 and NTB-A; however, other SLAMR such as CD229 may be also implicated as suggested from mice and human studies (47, 49). These pathways appear to be important in the recognition of EBV-infected B cells by T cells and in the activation of the T- and NK-cell cytotoxicity responses toward EBV-infected B cells. Another important pathway is dependent of the NKG2D receptor, well known to activate T- and NK-cell cytotoxic responses. In the absence of MAGT1, NKG2D expression on CD8^+^ T cells and NK cells is impaired leading to defective killing of EBV-infected B cells (79). Nevertheless, it would be interesting to know whether EBV-specific T cells expand normally in MAGT1-deficient patients as NKG2D has been also involved in the survival and expansion of CD8^+^ T cells during viral infections. The pair CD27–CD70 molecules forms a critical axis required for survival and expansion of EBV-specific T cells (91). The key role of T-cell expansion/proliferation to control EBV-infected B cells is also highlighted by the CTPS1 and RASGRP1 deficiencies, in which the capacity of T lymphocytes to proliferate in response to antigenic stimulation is specifically impaired.

Most of these deficiencies are characterized by a marked decreased or absence of invariant T cell populations iNKT and mucosal-associated invariant T (MAIT) cells, which raises the question of the role of these cells in immunity to EBV. iNKT cells are characterized by innate-like properties including prompt activation and production of large amounts of cytokines. These cells have been involved in a variety of immune responses including anti-viral immunity. Mice models have provided the evidence that SAP, ITK, and RASGRP1 are directly implicated in the development/homeostasis/function of iNKT cells (132–137). Analysis of XIAP-deficient patients also indicated that these cellular defects can be secondary to the EBV infection, leading to cell exhaustion in relation with the peculiar high sensitivity of iNKT and MAIT cells to AICD (138). There are only limited studies that assessed the role of iNKT cells in EBV infection, though it was shown that iNKT cells, in particular CD8^+^ iNKT cells have the capacity to directly lyse EBV-infected B cells expressing CD1d and to limit expansion of EBV-transformed B cells both *in vitro* and *in vivo* (139–141). However, RORC-deficient patients, who lack iNKT and MAIT cells are not particularly susceptible to EBV, indicating that these cells are not playing a critical role in immunity to EBV (142). Altered functions and decreased counts of NK cells are also often observed in a number of these immunodeficiencies. The role of NK cells might be particularly important during childhood, since KIR-negative early differentiated NK cells that expanded preferentially during IM and targeted lytically EBV-replicating B cells, progressively disappeared in the first decade of life (143). Recent data indicated that patients with severe combined immunodeficiency (SCID) who received bone marrow transplanted, although devoided of NK cells after immune reconstitution display an efficient immunity to EBV since none of them developed EBV-driven lymphoproliferation disorders, even 39 years after the transplantation (144). This suggests that NK cells are not essential for EBV immunity throughout the life. In any event, although they are not key components, accumulation of these different cellular defects might participate to the susceptibility to EBV in these genetic settings, particularly during childhood. CD4 T cell lymphopenia that is observed in several of these disease may also participate to EBV susceptibility, although the role of CD4^+^ T cells in the control of EBV infection is not clearly established (3). Arguing against a relevant role of CD4^+^ T cells, patients with MHC class II deficiency have a severe CD4 lymphopenia and do not develop EBV-driven LPDs and they have normal B cell counts. Although not really considered, some of these defects could also have intrinsic B-cell consequences that would further favor proliferation and/or lymphomagenesis of EBV-infected B cells in addition to the immune deficiency. In that respect, CD70 was shown to elicit reverse signaling involved in apoptosis of B cells and the recent study of MAGT1-deficient mice revealed a regulatory role of MAGT1 in B-cell development and proliferation that may explain the B lymphocytosis found in MAGT1-deficient patients (86, 145).

## Differential Diagnosis

Given the importance of the CD8^+^ T-cell response in immunity to EBV, it is not surprising that EBV-driven LPDs are also found in other PIDs associated with T-cell defects, but with a lower frequency. Herpes virus, in particular EBV, are often the trigger of HLH in patients with familial haemophagocytic lymphohistiocytosis (FHL), a group of diseases associated with impaired cytolytic activity of CD8^+^ T cells and NK cells. These diseases are caused by gene defects in the perforin gene and in the components of lytic granule exocytosis machinery (146). Patients with CVID or CID, particularly those affecting T-cell survival, migration, and F-actin mobilization in T cells, can also develop EBV-associated disorders (12). Those include, among others, deficiencies in NFκB1 (147), MST1/STK4 (148, 149), WASP (150), DOCK8 (151), GATA2 (152), and gain-of-function mutations in *PIK3CD* known to cause activated PI3K-delta syndrome (153). Patients with hypomorphic mutations in genes involved in T-cell development such as RAG1/2, DCLRE1C (ARTEMIS), or ZAP-70 can also experience EBV susceptibility (154–156). It should be considered that patients with the most severe T-cell defects will never present EBV problems since they develop very early-onset severe infections (other than EBV), requiring rapid bone marrow transplantation before they encounter EBV. At last, some of these immunodeficiencies (SCID) are also associated with a severe block in B-cell development, a cellular context that in all likelihood does not allow EBV infection establishment and dissemination.

## Therapeutics

So far, the only curative treatment for these PIDs is hematopoietic stem cell transplantation (HSCT). The first studies on large cohorts of XLP-1 and XLP-2 patients reported poor survival with or without HSCT (23, 157). This pejorative prognosis was partly linked to the use of full conditioning regimens associated with a high toxicity in patients (XIAP) and/or the absence of HLH remission at the time of HSCT. In the last few years, the use of reduced intensity conditionings and the development of new therapies like alemtuzumab (anti-CD52 antibody) has strongly improved the management of these diseases (33, 158, 159). Furthermore, rituximab (anti-CD20 antibody) has now a major role in the management of LPDs associated with these PIDs with patients being in remission after having received this treatment.

## Concluding Remarks

Molecular characterization of familial forms of EBV susceptibility has provided over the last 20 years, novel diagnostic tools for these disorders. Analysis of large cohorts and case reports have underlined non-EBV phenotypes associated with these PIDs such as IBD, pulmonary involvement, neurological disorders, or other typical infections (such as HPV), that can further help to the diagnosis. However, there are still a number of patients with a high susceptibility to EBV, in whom the molecular/genetic basis of their disease is not known and remains to be determined. In the light of the knowledge gained throughout the studies of the genetically determined forms of EBV susceptibility, we can speculate that these uncharacterized forms are caused by defects in molecules/components involved in T–B cell interactions and required for T-cell cytolytic responses and/or T-cell expansion.

## Author Contributions

SL wrote the manuscript and made the Figure 1. SW compiled data for Tables 1 and 2 and did the tables and participated to the writing of the manuscript.

## Conflict of Interest Statement

The authors declare that the research was conducted in the absence of any commercial or financial relationships that could be construed as a potential conflict of interest.
